# Mainstreaming traditional fruits, vegetables and pulses for nutrition, income, and sustainability in sub-Saharan Africa: the case for Kenya and Ethiopia

**DOI:** 10.3389/fnut.2023.1197703

**Published:** 2023-12-07

**Authors:** Peter Biu Ngigi, Céline Termote, Dominique Pallet, Marie Josèphe Amiot

**Affiliations:** ^1^MoISA, Univ Montpellier, CIHEAM-IAMM, CIRAD, INRAE, Institut Agro, IRD, Montpellier, France; ^2^UMR-Qualisud, CIRAD, Univ Montpellier, Avignon Université, Institut Agro, Université de La Réunion, Montpellier, France; ^3^Alliance Bioversity International and CIAT (Nairobi), Nairobi, Kenya

**Keywords:** malnutrition, poverty, inequality, agrobiodiversity, neglected-underutilized, priority species

## Abstract

This study documented existing knowledge on traditional fruits, vegetables and pulses in Kenya and Ethiopia. The aim was to identify neglected and underutilized species with high potential for food security, for their economic value and contribution to sustainable agriculture, based on a literature review and confirmation of existing data by local experts. In order of priority, the top 5 fruit species in Kenya are *Tamarindus indica L.*, *Adansonia digitata L.*, *Sclerocarya birrea (A.Rich.) Hochst*, *Balanites aegyptiaca (L.) Delile,* and *Ziziphus mauritiana Lam.,* for vegetables are *Amaranthus* spp., *Vigna unguiculata (L.) Walp*., *Solanum* spp., and *Cleome gynandra L*. Top fruits in Ethiopia are *Balanites aegyptiaca (L.) Delile*, *Ziziphus spina-christi (L.) Desf*., *Cordeauxia edulis Hemsl*., *Cordia africana Lam*., and *Mimusops kummel A. DC.,* for vegetables are *Brassica carinata A. Braun, Cucurbita pepo L*., and *Amaranthus* spp. In both countries, priority pulse species (no ranking) are *Phaseolus lunatus L*., *Sphenostylis stenocarpa (A.Rich.) Harms*, *Mucuna pruriens (L.) DC*., *Lablab purpureus (L.) Sweet,* and *Cajanus cajan (L.) Millsp*. Generally, these priority species are good sources of key nutrients known for their inadequate dietary intakes in sub-Saharan Africa, represent a safety net for household income, and contribute positively to ecosystem resilience in existing agricultural systems. Complete, accurate and reliable nutrient composition data are needed to raise consumer awareness about their nutritional and health benefits. Since women play a central role in traditional food systems, their empowerment, and hence resilience, increase the positive impact they can have on the households’ dietary diversity. In particular, introducing small-scale processing techniques and marketing strategies could enhance their supply and consumption.

## Introduction

### Traditional/indigenous food plants: neglected or underutilized

Indigenous food plants are plants that have evolved naturally in a specific bio-region in conjunction with those that were introduced into the region and adapted so well that they have become an integral part of the local food culture (1). The term “traditional food plants” is often used to indicate that the plants have been consumed in a region for several centuries (2). What is important is not so much the terminology used but the fact that the modes of cultivation, collection, preparation, and consumption of the food plants are deeply embedded in local cuisine, culture, folklore, and language (FAO, 2001) (3). For the purpose of this study, the term “traditional food plants” is thus used.

Many traditional food plants are not included in local and national diets and consequently remain either neglected or underutilized. They are termed “neglected” if they are grown primarily in their centers of origin or centers of diversity by traditional farmers, where they remain important for the subsistence of local communities, and they are termed “underutilized” if they were once more extensively grown but have fallen into disuse for a variety of agronomic, genetic, economic, and cultural reasons (4, 5). In either case, these traditional food plants have received little or no attention from agricultural researchers, plant breeders or policy makers. Data on their production, consumption, nutrition composition and trade value are therefore largely decentralized, unavailable, incomplete and hence limiting, and often limited among indigenous communities (6).

### Inadequate or declining consumption of fruits, vegetables, and pulses

**Figure 1:**
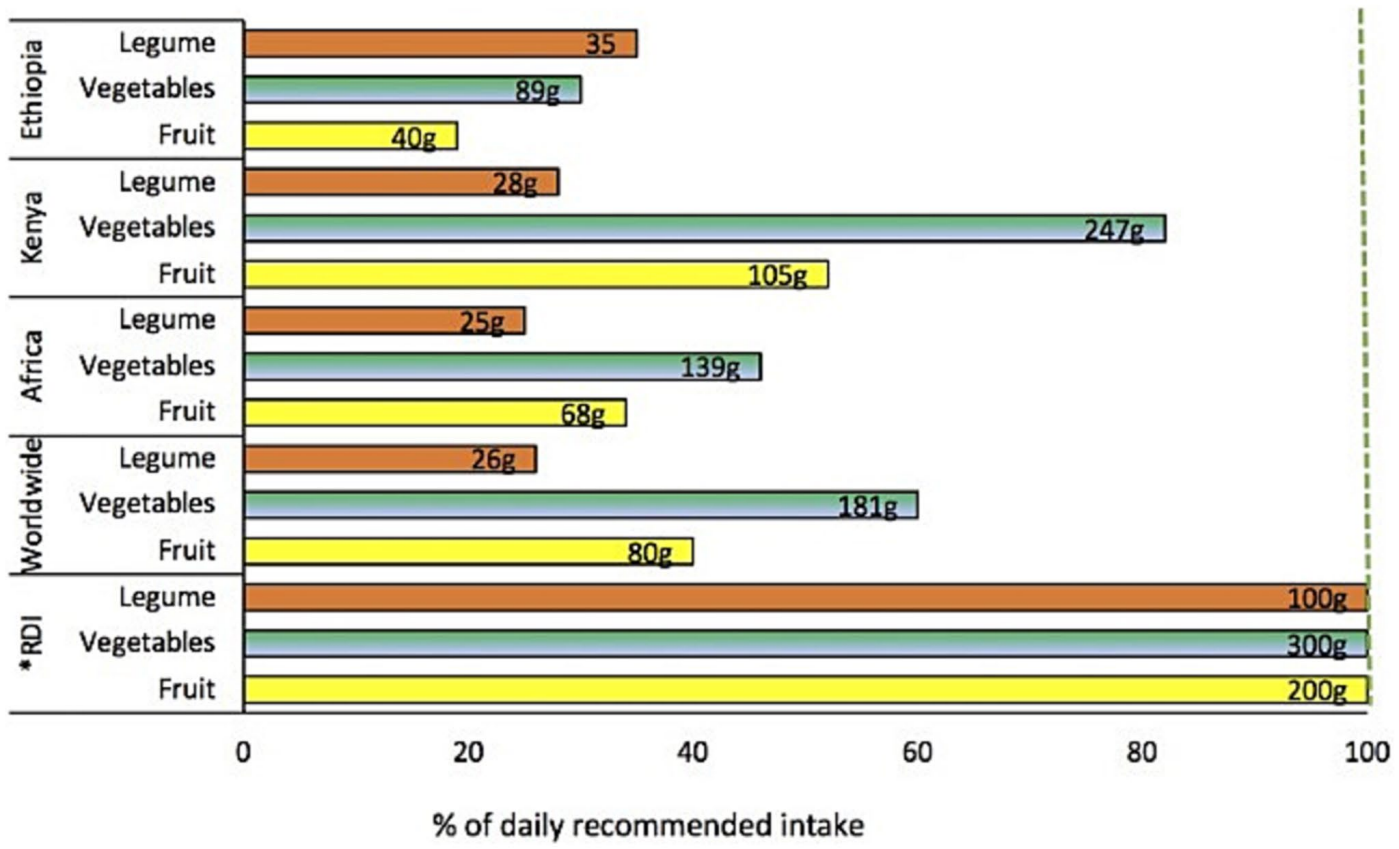
Intakes of fruits, vegetables, and legumes as a percentage of *DRI, Daily recommended intake by EAT-Lancet Commission (16). Data source: Global Dietary Database 2019 (17).

Fruits, vegetables, and pulses form the basis of dietary diversity (7), particularly as the primary sources of important vitamins, minerals, protein and fibers, which are insufficiently supplied and whose dietary intakes are consequently often inadequate in sub-Saharan Africa (SSA) (8, 9), where the consumption of fruits, vegetables, and pulses is either insufficient or declining (10, 11). In SSA, food accounts for a very large proportion of the household budget, for example, for 56% in Ethiopia, and 58% in Kenya (12, 13). During the period 2017 to 2019, the cost of a healthy diet in Africa underwent the highest increase (13%) in the world (14). To alleviate hunger, households are forced to prioritize staple foods while more expensive nutrition-dense food such as fruits, vegetables, and pulses are considered unaffordable. According to Harris et al. (15), poor availability, accessibility, affordability, and preferences affect the inclusion of fruits, vegetables, and pulses in household diets and there is consequently an enormous deficit in the consumption of these foods in SSA (Figure 1). For a healthy daily adult diet representing 2,500 kcal, the EAT-Lancet Commission recommends a daily intake of 300 g of vegetables, 200 g of fruit, and 100 g of legumes of which half (50 g) should be pulses (16). To obtain the health and nutrition benefits of fruits and vegetables it is recommended to consume at least 400 g each day or 5 servings of 80 g each (18, 19). Moreover, fruits, vegetables, and pulses should be consumed in different varieties and if possible, above the recommended amounts (20). Exploiting the large number of largely under-used traditional plants with edible fruits, shoots, leaves, flowers, and seeds of high nutritional and economic value could help reach this goal (21).

The present study focuses on neglected and underutilized species with a high potential for food security, for their economic value and their contribution to sustainable agriculture. To achieve this goal, priority species should: (i) fill the nutrient gaps in diets (protein, fibers, iron, zinc, vitamin A) in order to have a greater impact on public health, (ii) increase agricultural income to enable the purchase of a diversity of foods, as well as be consumed by the household if not completely sold, (iii) simultaneously address the nutritional, agricultural and environmental dimensions of the malnutrition and poverty issues, and (iv) account for cultural preferences, social pressures, women’s resilience and other elements related to human behavior.

## Materials and methods

First, a list of available species of traditional fruit trees, leafy vegetables, and pulses in Ethiopia and Kenya was created based on existing plant databases and was confirmed by local experts. The aim was to fill the gap concerning easily accessible country-specific species, particularly using accepted scientific names in the World Flora Online (WFO) database (22). The rich biodiversity of traditional food plants in the two countries was apparent at first glance. The species list that was generated a great help in tackling the nomenclature confusion in the literature caused by use of different scientific synonyms for the same plant species. This documentation database also enabled identification of floristic regions (Ethiopia) or agroecological zones (Kenya) with the highest abundance of traditional fruit trees, leafy vegetables, and pulses (23).

Since not all traditional fruits, vegetables, or pulses are equally attractive for nutritional, economic and agronomic reasons, several criteria were used to identify, rank, select and prioritize species based on their nutritional, economic and agronomic traits, among other benefits. Based on literature review, existing scientific and indigenous knowledge of traditional fruits, vegetables and pulses was documented and was confirmed by local experts from Kenya and Ethiopia. In this study, the primary selection criterion of the top 5 species was food security and economic potential; the second selection criterion was contribution to ecosystem resilience. Candidate plant species were identified based on their unique nutritional, economic, and agronomic attributes. For traditional fruit trees, the frequency of being included among the top priority 5 species in past prioritization studies informed a mutual decision on the most potential species. The ranking criteria used in past prioritization studies and respective references are presented in Appendices 1–3. For traditional leafy vegetables, very few prioritization studies are available and no ranking is reported. The top 5 most frequently prioritized species and references are listed as presented in Appendix 5. For pulses, no prioritization studies are available. In this case, past research attention was used under which, pulse species that have received the least research attention but are characterized by multipurpose services are listed as presented in Appendix 6.

## Findings

Five traditional fruit trees are frequently and consistently selected among the top 5 priority species in Kenya (listed in Table 1; Appendix 1) (21, 24–28). In order of priority, these include *Tamarindus indica L*., *Adansonia digitata L*., *Sclerocarya birrea (A.Rich.) Hochst*, *Balanites aegyptiaca (L.) Delile,* and *Ziziphus mauritiana Lam*. Other important traditional fruit trees but less frequently ranked among the top 5 priority species include *Ximenia americana L*., *Berchemia discolor (Klotzsch) Hemsl*., *Ancylobothrys tayloris (Stapf) Pichon,* and *Vitex doniana Sweet*. In Ethiopia, the top 5 priority species include, *Balanites aegyptiaca (L.) Delile*, *Ziziphus spina-christi (L.) Desf*., *Cordeauxia edulis Hemsl*., *Cordia africana Lam*., and *Mimusops kummel A. DC*. (listed in Table 1; Appendix 2) (24, 25, 29–31). Other important priority species ranked among the top 5 species include *Sclerocarya birrea (A. Rich.) Hochst*., and *Vitellaria paradoxa Gaertn*. There has been no systematic prioritization study covering the entire African continent due to its diverse ecology, farming systems, and vast geographical area. However, based on the extent of their market and preferences, the priority fruit trees that are available across regions of the continent include *Adansonia digitata L*., *Tamarindus indica L*., *Ziziphus spina-christi (L.) Desf*., *Sclerocarya birrea (A. Rich.) Hochst*., and *Balanites aegyptiaca (L.) Delile* as listed in Table 1 and Appendix 3. The specific selection criteria used for priority traditional fruit trees are summarized in the respective appendices.

**Table 1:** Top 5 traditional fruit trees (in order of priority) in Kenya, Ethiopia, and other African regions.

Country	*Scientific name*	Common name	References
Kenya	*Tamarindus indica L.*	Tamarind	(21, 24–28)
	*Adansonia digitata L.*	Baobab	
	*Sclerocarya birrea (A.Rich.) Hochst*	Marula	
	*Balanites aegyptiaca (L.) Delile*	Desert date	
	*Ziziphus mauritiana Lam.*	Ber	
Ethiopia	*Balanites aegyptiaca (L.) Delile*	Desert date	(24, 25, 29–31)
	*Ziziphus spina-christi (L.) Desf.*	Jujube	
	*Cordeauxia edulis Hemsl.*	Yeheb	
	*Cordia africana Lam.*	-	
	*Mimusops kummel A. DC.*	-	
**Region: East Africa (EA), Sahelian zone (SZ), South Africa (SA), West Africa (WA)**			
EA, SZ, SA	*Adansonia digitata L.*	Baobab	(32–36)
EA, SZ	*Tamarindus indica L.*	Tamarind	
SZ, SA	*Ziziphus spina-christi (L.) Desf.*	Jujube	
EA, SA	*Sclerocarya birrea (A. Rich.) Hochst.*	Marula	
EA, WA	*Balanites aegyptiaca (L.) Delile*	Desert date	

Traditional leafy vegetable species in Kenya and Ethiopia were selected based on their economic and nutritional potential. In Kenya, the top 5 species are *Amaranthus* spp., *Vigna unguiculata (L.) Walp*., *Solanum* spp., and *Cleome gynandra L* (listed in Table 2; Appendix 5) (37, 38). In Ethiopia, they include *Brassica carinata A. Braun*, *Cucurbita pepo L*., and *Amaranthus* spp. (listed in Table 2; Appendix 5) (29–31). The top 10 most cited priority traditional leafy vegetables in Kenya and Ethiopia are listed in Table 3. These species form a basis for future prioritization studies.

**Table 2:** Top 5 traditional leafy vegetables (in order of priority) in Kenya and Ethiopia.

Country	*Scientific name*	Selection criteria	References
Kenya	*Amaranthus* spp. (Amaranth)	Economic and nutritional potential	(37, 38)
	*Vigna unguiculata (L.) Walp.* (Cowpeas leaves)		
	*Solanum* spp. (African nightshade)		
	*Cleome gynandra L.* (Spiderplant)		
Ethiopia	*Brassica carinata A.Braun* (Abyssinian Cabbage)	Availability, palatability, market, medicinal	(29–31)
	*Cucurbita pepo L.* (Pumpkin leaves)		
	*Amaranthus* spp. (Amaranth)		

**Table 3:** Top 10 priority leafy vegetable species most frequently cited for research and promotion in Kenya and Ethiopia.

	*Scientific name* (common name)	Selection criteria	References
Kenya	*Amaranthus blitum L*. (Amaranthus)	Nutritional and economic potential, indigenous knowledge of production, agronomic, and cultural practices	(5, 37, 39, 40)
	*Vigna unguiculata (L.) Walp.* (Cowpea leaves)		
	*Solanum villosum Mill./scabrum Mill.* (African nightshade)		
	*Cleome gynandra L.* (Spiderplant)		
	*Cucurbita pepo L.* (Pumpkin leaves)		
	*Corchorus olitorius L./tricularis L.* (Jute mallow)		
	*Crotalaria ochroleuca G.Don/brevidens Benth.* (Slenderleaf)		
	*Launaea cornuta (Hochst. ex Oliv. & Hiern) C.Jeffrey* (Bitter lettuce)		
	*Abelmoschus esculentus (L.) Moench* (Okra)		
	*Urtica dioica L.* (Stinging nettle)		
Ethiopia	*Brassica carinata A.Braun* (Abyssinian Cabbage)	Distribution, Seasonality, user preferences, abundance	(41–44)
	*Brassica oleracea L.* (Chinese Kale)		
	*Corchorus trilocularis L.* (Jew’s mallow)		
	*Balanites aegyptiaca (L.) Delile* (Desert dates)		
	*Moringa stenopetala (Baker f.) Cufod.* (Cabbage tree)		
	*Haplocarpha schimperi (Sch. Bip.) Beauv.* (Onefruit)		
	*Urtica dioica L.* (Stinging nettle)		
	*Beta vulgaris L.* (Beet)		
	*Coccinia abyssinica (Lam.) Cogn.* (Anchote)		
	*Erucastrum arabicum Fisch. & C.A.Mey.*		

As there have been no prioritization studies on pulses in Kenya and Ethiopia to date, available traditional pulse species were categorized based on past research on (1) mainstreamed pulses that have been the subject of significant research and are the most widely grown and consumed, (2) neglected, underutilized, and promising future pulses that adapt particular well to environments and provide multipurpose services, (3) neglected and underutilized pulses for mainstreaming into agri-food systems, (4) other potential neglected and underutilized pulses (Table 4). The category of neglected, underutilized, and promising future pulses that display particular adaptation to the environment and provide multipurpose services are the most in line with the primary and secondary selection criteria for the top 5 priority species in this study. Based on multi-purposes, nutrient value, market prospects, and their contribution to ecosystem resilience, the top 6 priority long-life cycle pulse species for future research and promotion include *Phaseolus lunatus L.*, *Sphenostylis stenocarpa (A.Rich.) Harms*, *Mucuna pruriens (L.) DC.*, *Lablab purpureus (L.) Sweet*, and *Cajanus cajan (L.) Millsp.* (listed in Table 5; Appendix 6). Although *Cajanus cajan (L.) Millsp*. has been the subject of considerable research, its multipurpose use is largely underexploited and in particular, its properties that enhance soil nutrient content. All six pulse species in this category were thus considered as priority in both countries.

**Table 4:** Categories of pulse species based on past research, current research gaps, and future research opportunities.

Pulse category	*Scientific name* (common name)	Research attention	Reference
Mainstreamed pulses with significant research attention and promotion	*Phaseolus vulgaris L.* (Common bean)	312 improved varieties released	(45, 46)
	*Vigna unguiculata (L.) Walp.* (Cowpea)	169 improved varieties released	
	*Cajanus cajan (L.) Millsp.* (Chickpea)	>48 improved varieties released	
	*Cicer arietinum L.* (Pigeon pea)	21 improved varieties released	
Neglected, underutilized pulses, adaptation to environments, and provide multipurpose services	*Phaseolus lunatus L.* (Lima bean)	Limited research investment; limited literature available but many research needs	(46, 47)
	*Sphenostylis stenocarpa (A.Rich.) Harms* (African yam bean)		
	*Mucuna pruriens (L.) DC.* (Velvet bean)		
	*Lablab purpureus (L.) Sweet* (Lablab bean)		
Neglected and underutilized pulses for mainstreaming into agri-food systems	*Macrotyloma geocarpum (Harms) Marechal & Baudet* (Ground bean)	Limited literature available but many research needs; under-researched or orphan African food crops	(46, 47)
	*Vigna subterranea (L.) Verdc.* (Bambara groundnut)		
	*Tamarindus indica L.* (Tamarind)		
Other potential neglected and underutilized pulses	*Canavalia ensiformis (L.) DC.* (Jack bean)	No information available on research undertaken to date; limited information on improvement of plant type	(48)
	*Macrotyloma uniflorum (Lam.) Verdc.* (Horse gram)		
	*Parkia biglobosa (Jacq.) G.Don* (African locust bean)		
	*Vicia faba L.* (Faba bean)		
	*Detarium microcarpum Guill. & Perr.* (Sweet detar)		
	*Vigna radiata (L.) R.Wilczek* (Mung bean)		
	*Lathyrus sativus L.* (Grass pea)		
	*Lens culinaris Medik* (Lentil)		
	*Lupinus albus L.* (White lupin)		
	*Trigonella foenum-graecum L.* (Fenugreek)		
	*Detarium senegalense J.F.Gmel.* (Ditax)		
	*Dialium guineense Willd.* (Velvet tamarind)		

**Table 5:** Nutrition, income, and ecosystem resilience potential of underutilized and neglected long-life cycle pulses.

Pulse	Edible parts	Key nutrients	^*^Uses	^*^Resilience	Market prospects
*Phaseolus lunatus L.* (Lima bean)	Seed, pod, leaf	Protein, fiber, Vitamin B1, B2, B3, B6, B9, K, E, Ca, Fe, Mg, P, K, Na, Zn	a, b, c, d, i	1, 2, 3, 6, 7, 8	Rank 2nd after *P. vulgaris* in economic interest
*Cajanus cajan (L.) Millsp.* (Pigeon pea)	Seed, pod, leaf, shoot	Protein, fiber, vitamin A, C, B1, B2, B3, B6, B9, Fe, Zn, Cu, Ca, P, Mg, Na, K	a, b, c, d, e, f, g	1, 4	Large market and demand for processed products
*Sphenostylis stenocarpa (A.Rich.) Harms* (African yam bean)	Seed, pod, leaf, root	Protein, fiber, vitamin C, B1, B2, B3, B6, Ca, Fe, Mg, P, K, Na, Zn, Mn, Se	a, b, d	1, 9	Most economically important species in the genus
*Mucuna pruriens (L.) DC.* (Velvet bean)	Seed, pod	Protein, fiber, vitamin B1, B2, K, Ca, Fe, Mg, P, K, Na, Zn, Cu, Mn	a, b, c, d, e, f, g, h	1, 7, 8	Productivity better than most cover crops
*Lablab purpureus (L.) Sweet.* (Lablab bean)	Seed, pod, leaf, flower, tuber	Protein, fiber, vitamin B1, B2, B3, B6, B9 Minerals Cca, Fe, Mg, P, K, Na, Zn	a, b, c, d, f, g, h	1, 2, 6, 7	More valuable in terms of price than common bean
*Canavalia ensiformis (L.) DC*. (Jack bean)	Seed, pod, leaf	Protein, fiber, Ca, Fe, Mg, P, K, Na, Zn, Cu, Mn	a, b, c, d, g, f, h, i, j	1,4, 5, 7, 8	High market acceptance and higher market prices

^*^Uses: (a) food, (b) medicines, (c) fodder, (d) high ability to fix nitrogen, (e) enhances P availability, (f) green manure, (g) cover crop, (h) weed control, (i) nematode control, (j) pest, and disease control. ^*^Resilience: (1) drought tolerance, (2) high temperature tolerance, (3) soil toxicity tolerance, (4) acidic soil tolerance, (5) adapted to nutrient-depleted highly leached soils, (6) adaptable to adverse environments (7) pest resistance, (8) disease resistance, (9) pesticidal potential, (10) deep root system. The uses and resilience list is random, not ranked in order of importance nor is it applicable across African regions and countries nor within countries. Information sources are listed in Appendix 6.

**Table 6:** Nutrition, income, and ecosystem resilience potential of underutilized and neglected priority fruit trees.

Fruit tree	Edible parts	Key nutrients	^*^Uses	^*^Resilience	Market prospects
*Tamarindus indica L.* (Tamarind)	Leaves, fruit pulp, seeds	Protein, vitamin C, B, ß-carotene	a, b, c, d, h, j, l, f, m, o	1, 2, 3, 4	Fruit pulp → market value product
*Balanites aegyptiaca (L.) Delile* (Desert date)	Leaves, flowers, fruit pulp, seeds	Protein, lipids, vitamin C, B, Fe, Zn	a, b, c, d, e, h, i, j, f, m, r	1, 2	Local market: leaves, fruits, nuts International market: drug manufacturing
*Adansonia digitata L.* (Baobab)	Fruits, shoots, leaves	Vitamin A, C, E, B1, B2, B3, protein, Fe, Ca, Mg, Zn, P, K, fiber, ß carotene	a, b, c, d, i, n, s	2, 4	Every part of the tree has a market value. Various products approved as “novel food” by the European Commission
*Sclerocarya birrea (A.Rich.) Hochst.* (Marula)	Fruit pulp, seeds	Protein, lipids, vitamin C, B_1_, Fe, Zn	a, b, c, d, i, j, f	1, 2, 4	Food industry, cosmetics industry, biodiesel products
*Ziziphus spina-christi (L.) Desf.* (jujube)	Fruits		a, b, c, d, p, j, f, q	1, 2, 3, 4	Food industry

^*^Uses: (a) food, (b) income, (c) fodder, (d) medicine, (e) cosmetics, (f) fuelwood, (g) bio-pesticides, (h) nitrogen fixing, (i) bio-insecticides, (j) wood, (k) processing, (l) food preservatives, (m) soil protection, (n) green manure, (o) weed control, (p) nematode control, (q) increase available phosphorous, (r) bio-diesel, (s) soap making. ^*^Resilience: (1) Highly adaptable to Sahelian soil types, (2) drought tolerant, (3) resistant to heat, (4) adaptable to adverse climatic conditions. The uses and resilience list is random, not ranked in order of importance nor applicable across African regions and countries nor within countries. Information sources are listed in Appendix 4.

The above priority traditional fruit trees, leafy vegetables, and pulses are usually described as having several household uses that could help fill existing nutritional and economic gaps, cultural needs and preferences, as well as contribute to sustainable agriculture through ecosystem resilience (Tables 5–7; Appendices 4–6). It should be noted that multiple uses are listed randomly as they were in the literature and are therefore not ranked in order of importance, nor is the listing universally applicable across African regions and countries or even within countries. The priority species provide edible fruits, shoots, leaves, flowers and seeds that are good sources of important vitamins and minerals whose dietary intake is often inadequate in SSA. Moreover, both food and non-food food products rank high economically and could therefore serve as a safety net by enhancing household purchasing power for food items that are not included in local subsistence production. Priorities are also climate resistance, local availability, adaptability to arid and semi-arid conditions, compatibility with other crops, and capacity to contribute to ecosystem resilience and sustainable agriculture. They are potential candidates for inclusion in existing farming system in the drylands of SSA which are usually characterized by limited agrobiodiversity.

**Table 7:** Nutrition, income, and ecosystem resilience potential of underutilized and neglected leafy vegetables.

Leafy vegetable	Edible parts	Key nutrients	^*^Uses	^*^Resilience	Market prospects
*Amaranthus blitum L.* (Amaranthus)	Leaves, seeds	Leaves: A, C, B1. Ca, Fe, carotene, folate, vitamin C. Seed: protein (lysine and methionine) fiber, K, Ca, P, vitamin A, C	a, b, c, e, k, l, m, n, o	a, b, d, e, g	Seed flour for baking industry—gluten free; seeds malted for beer
*Cleome gynandra L.* (Spiderplant)	Leaves, shoots	Vitamin A, C, E, Ca, Fe, Zn, Mg, β-carotene, protein	a, b, c, d	b, c, d, e	Ready rural and urban market; grows very fast; the only vegetable available during relish-gap period
*Corchorus olitorius L./tricularis L.* (Jute mallow)	Leaves, fruits (okra)	Vitamin A, C, E, K, Ca, Mg, Fe, β-carotene, protein	a, b, e, j, m, p, q	a, e, f, j	High market value, consumer preference, and nutritional value
*Crotalaria ochroleuca G.Don /brevidens Benth.* (Slenderleaf)	Leaves, shoots, flowers	Vitamin C, β-carotene, B1, B2, B3, protein, Fe, Ca	a, b, c, d, f, g, h, i, j, r	a, d, h, i	Increased demand in local and regional markets
*Launaea cornuta (Hochst. ex Oliv. & Hiern) C.Jeffrey.* (Bitter Lettuce)	Leaves	Protein, fiber, vitamin C, Na, K, Ca, Fe, P	a, b, c, d	g	Ready urban market; local availability; grows naturally; low input requirements

^*^Uses: (a) food, (b) medicines, (c) fodder, (d) insecticides, (e) cosmetics, (f) green manure, (g) cover crop, (h) striga control, (i) nematode control, (j) fiber, (k) beer, (l) laundry starch, (m) paper making, (n) films, (o) dye, (p) soap, (q) waxes, (r) ornamental plant. *Resilience: (a) adaptation to different environments, (b) heat resistance, (c) cold resistance, (d) drought resistance, (e) pest resistance, (f) disease resistance, (g) adaptable to marginal soils, (h) nitrogen-fixing ability, (i) nematode control, (j) can grow where no other foliage crops can grow. The uses and resilience list is random, not ranked in order of importance, nor applicable across African regions and countries nor within countries. Information sources are listed in Appendix 5.

## Discussion

SSA contains a large number of under-exploited traditional plant species that are of high nutritional value and could therefore play a significant role in diversifying diets at local, national and international level (21). Both the food and non-food products of these species have high market value that could diversify sources of household income and hence, access to food markets. Some traditional food plants are often available in periods when most of the food stocks are used up and new crops are not ready to harvest. As a result, their consumption increases as stocks of staple crops decline and hence, they play an important nutritional and economic role in filling seasonal gaps (26, 49). Traditional food plants could also contribute positively to the ecosystem resilience of existing farming systems by providing green manure, nitrogen-fixing abilities, increasing soil phosphorous availability, and helping control soil erosion, parasitic weeds, nematodes, pests and diseases. These plants are also well adapted to diverse and contrasting climates, cold or heat, droughts or floods, and poor quality soils, and also show good resistance to pests and diseases (50). Compared to large-scale conventional staple crops, most under-exploited traditional food plants are available locally, easy to grow, require simpler technologies and fewer inputs of fertilizer and pesticide and hence, are less damaging to the environment while addressing the cultural needs of indigenous society (11). Integrating the wealth of diverse traditional food plants into local food systems thus has great potential (20), and given the current coexisting issues of malnutrition, climate change, and degradation of agricultural soils in SSA, traditional food plants have the potential to create new value chains provided there is a significant increase in the necessary research and development (51). The nutritional, economic and agronomic benefits of their production make them attractive components of existing food and farming systems, and growing them could enhance agrobiodiversity, sustainable agriculture, ecosystem resilience, food and nutritional security, and economic development (11).

As a first step toward integrating traditional food plants for food and nutritional security, income improvement, and agricultural sustainability, it is essential to identify the most valuable species for smallholder farmers, consumers and the market. The priority traditional food plants identified are neglected or underutilized in the two study countries, which means that, data on production, consumption and the preferences of farmers and/or consumers are either lacking or largely decentralized in the literature or are unpublished indigenous knowledge. Plants are underutilized due to low and irregular production which is affected by a preference for conventional staple cereal crops (52) or neglected due to lack of reliable data to evaluate their contribution to the diet or economy at local, national and international levels (1). These limitations complicate the assessment and identification of priority species for inclusion in existing farming and food systems. The distribution and use of these species also varies between and within countries in line with variations in ecology, farming systems and climatic conditions. As a result, the priority traditional food plants identified in Kenya and Ethiopia are not the same and variation in priority species and ranking is expected across floristic regions or agroecological zones. Despite these challenges, existing knowledge of the identified priority traditional fruit trees, leafy vegetables and pulses was documented and highlighted their potential to contribute to sustainable food security, livelihoods, and agriculture.

The priority species are characterized by high nutritional value and could play an important role in overcoming existing deficits in fruit, vegetable and pulse consumption and consequently increase intakes of key nutrients. In resource-poor areas, these traditional foods are often the only local and affordable alternative sources of protein, fiber, vitamins and minerals. Local production makes them affordable particularly in rural drylands where the majority of poor rural populations live. This is one of the main reason why they are still consumed (53). They are also kept in farming systems because of their social-cultural significance and easy use (1). The fact they are native means knowledge, expertise, skills, and production processes still exist among the indigenous communities (52). However, these traditional foods are not widely used due to misperceptions that they are inferior to conventional staple foods and hence, considered obsolete and unworthy of research and development (11, 54). A major contributing factor is the lack of consumer awareness about their nutrition and health benefits. Nutrition education is therefore paramount in guiding consumer preferences and choices and hence, adoption by both the farmers and markets. Yet, data on nutrient content particularly concerning neglected and underutilized traditional food plants is either unavailable, incomplete or unreliable. The available data on nutrient content in the literature and in food composition tables vary considerably. According to Termote et al. (55), this may be due to the use of different sampling and analytical methods on the one hand, and the geographical and natural variability of nutrient composition and concentrations that occurs among varieties of the same species, on the other hand. This is particularly true of undomesticated species. In the current study, it was therefore considered impractical to compare and rank priority traditional food plants based on their nutrient content or on their contribution to recommended daily intakes. Instead, the major nutrients supplied by the species are reported simply to highlight their nutritional value as presented in Appendices 4–6 for fruits, leafy vegetables, and pulses respectively, under “Nutrient value.” However, there is a real need to assess the nutritional composition of neglected and underutilized traditional foods. Complete, accurate, reliable and representative data on the nutritional composition of traditional foods is critical for raising consumer awareness and hence, increasing consumption.

Priority traditional food plants are also characterized based on their income generating potential. Depending on the species, they may rank high in market value either locally, regionally or internationally (56). A ready market for both food and non-food products therefore exists, but due to lack of modern industrialized markets, there has been little social and scientific research on—or investment in—the benefits and use of these foods (57, 58). The adoption of the priority species by farmers for cultivation or to safeguard existing populations of species remain low unless economic returns on investment associated with their production are profitable (59). If developed successfully, traditional value-added food products could occupy a market niche at different market levels and increase different sources of income for households, smallholder farmers and vulnerable groups, in particular women in rural drylands. This would contribute to the year-round supply and consumption of a diversified diet both through subsistence production and income-generating pathways.

In general, stable access to and the stable quality of food is linked to resilience (60). The priority traditional food plants identified in the current study could play a critical role in the agroecological resilience of existing farming systems. The plants are characterized by adaptability to the diverse biotic and abiotic conditions of SSA drylands. However, due to increase in human and livestock populations in SSA, today these priority traditional food plants are under enormous pressure that has led to intensive species degradation and losses (52). To reverse this situation, it is possible to include them in existing farming systems (33). On one hand, this would expand fruit, vegetable, and pulse production in SSA, which is currently mainly restricted to high altitude regions with moderate to adequate rainfall. On the other hand, their management either in the wild or cultivated on-farm would benefit the environment as they are less reliant on agrochemical inputs, help stabilize agroecosystems, enhance biodiversity and contribute to carbon sequestration.

Agriculture is the most important economic activity in SSA. It contributes to dietary diversity through agrobiodiversity and/or income-generating pathways. Currently, conflicts, climate variability and extreme climate events, economic slowdown and downturn adversely affect agricultural outputs (14). Healthy diets in SSA are unaffordable because, on one hand, the drivers of agricultural production push up the cost of fruits, vegetables, and pulses beyond what the poor population can afford. On the other hand, poverty is exacerbated by inequalities based on gender, youth, ethnicity, and disability (61, 62). With insufficient production and often little agrobiodiversity in farming systems, the low economic status of households adversely influences access to food in terms of quality and diversity. Inequality and poverty are common denominators for factors associated with widespread food insecurity and malnutrition (63). Among the adaptive strategies for food security in SSA drylands in which women are the most actively involved, are gathering of wild species, cultivation, harvesting, processing and sale of traditional fruits, vegetables and pulses (64). Traditional food cultures are informed by locally-specific social relations in which women often play a central role (14). Women are decision makers concerning household food consumption (64, 65). According to Jones et al. (66), women’s control of income and decision making has significant benefits for nutrition outcomes at the child and household levels. However, women in SSA are also the most affected by inequality and the poverty trap. They are disproportionately and adversely affected in terms of economic opportunities and access to nutritious foods (14). Increased access to productive resources, technologies and innovation in exploiting traditional food crops would both improve women’s resilience (36), and help incorporate neglected and underutilized traditional food plants. Access to traditional fruit trees, leafy vegetables, and pulses could reduce the likelihood that female-headed households face food and nutritional insecurity (64). In SSA, the harvesting, processing and trading of fruits, leafy vegetables, and pulses is primarily performed by women. Women are often deeply involved in and benefit from the production, processing and sale of traditional foods (65). This may explain why agricultural biodiversity is more positively associated with female-headed households than with male-headed households (66). However, women’s food and nutritional security is deeply affected by their lack of financial independence. Considering that agricultural income controlled by women has a greater positive impact on household dietary diversity than income controlled by men (67), income control and decision making by women have major advantages for child and household-level nutrition outcomes. This is because women often purchase foods and other health-related inputs that directly benefit the health and nutritional status of the members of their household. Women’s different purchasing behavior, as well as their selection and use of priority traditional food plants could reinforce women’s empowerment as a factor in changing the relationships between agricultural biodiversity, dietary diversity and health.

There is therefore a need to explore suitable processing techniques and culinary practices for traditional fruits, leafy vegetables and pulses. Food processing could tap the unexploited but high potential of traditional foods to provide employment opportunities, the development of small and medium-sized enterprises and generate income (68). As a first step, adequate postharvest handling techniques are needed to reduce post-harvest losses and maintain the quality of the plant products. In particular, small-scale processing techniques that involve women could help extend the shelf life and consequently increase the consumption, availability and access to traditional foods (36). In addition, biological or thermal food processing techniques can reduce antinutritional compounds and improve digestibility of these foods and bioavailability of some nutrients. As mentioned above, traditional foods and products play a critical role for women for whom they represent safety nets as marketable goods that enable them to buy food for their households (64). To ensure sustainability, a marketing strategy is needed to be sure that processed food products do not cost more than vulnerable population groups can afford as well as to avoid failure in promoting the consumption of traditional foods.

## Conclusion

The top priority species reported in this study are characterized by multiple uses, they are good sources of nutrients whose dietary intakes are currently often inadequate in SSA, they are ranked high economically and could therefore serve as safety-nets for household incomes, and contribute to sustainable agriculture through ecosystem resilience. However, traditional food plants are perceived as slow growing and as producing low yields and therefore as inappropriate for cultivation. This perception has been aggravated by the limited understanding of their roles in nature in terms of variability, reproductive biology and propagation as well as the absence of techniques for adding value and for facilitating their cultivation. As a result, the true extent to which traditional food plants can alleviate food insecurity remains unknown. Neglected and underutilized traditional food plants should therefore be included in future dietary surveys and their nutritional profiles specified in order to (i) assess their capacity to fill the gaps in (micro) nutrients and (ii) quantify the supply and transform it into production. Food composition analysis in also needed to provide missing data on their nutrient content, to reduce existing variability of available data and to raise consumer awareness. Small-scale processing techniques particularly those that involve women are also needed along with marketing strategies to avoid failures as a result of expensive value-added food products. To ensure the acceptability and adoption of traditional food plant species, consensus is needed among different stakeholders. In particular, the needs of communities and the points of view of researchers must be reconciled. Although this study concerns only two African countries, it has a general scope in SSA and beyond, where numerous plant species studied are present or food by-products are consumed.

After decades of research neglect and hence underutilization of traditional food plants, their contribution to the diet, income and ecosystem services remains largely unevaluated. The available data is poorly managed, highly fragmented and scattered across past decades. Recent data sources on nutritional, economic, and resilience potential are either lacking or only published earlier than 5 years ago and hence, it was necessary to include these data sources. This is a major constraint, characteristic to neglected and underutilized traditional food plants that highlights the research gap yet further. Besides centralizing existing knowledge, this study also calls for future research priorities in mainstreaming these superior traditional food plants to achieve multiple sustainable development goals.

## Author contributions

NPB wrote the first draft of the manuscript. All authors contributed to conception, design of the study, manuscript revision, read, and approved the submitted version.
