# Supplementary material for: Mainstreaming traditional fruits, vegetables and pulses for nutrition, income, and sustainability in sub-Saharan Africa: the case for Kenya and Ethiopia
Source: Front Nutr. 2023 Dec 7;10:1197703. doi: 10.3389/fnut.2023.1197703 (PMC10749635; doi:10.3389/fnut.2023.1197703)
Supplement: Supplementary file 1 [file Data_Sheet_1.docx]

***Supplementary Material***

**Mainstreaming traditional fruits, vegetables and pulses for nutrition, income, and sustainability in sub-Saharan Africa: the case for Kenya and Ethiopia**

*Peter Biu NGIGI^1, 2*^, Celine TERMOTE^3^, Dominique PALLET^2^, Marie Josèphe AMIOT^1^

^1^MoISA, Univ Montpellier, CIHEAM-IAMM, CIRAD, INRAE, Institut Agro, IRD, Montpellier, France, ^2^UMR-Qualisud, CIRAD, Univ Montpellier, Avignon Université, Institut Agro, Université de La Réunion, Montpellier, France, ^3^Alliance Bioversity International and CIAT (Nairobi), Kenya

***Correspondence**: [peterbiu@outlook.com](mailto:peterbiu@outlook.com)

1. **Supplementary Tables**

**Appendix 1**: Priority ranking of traditional fruit trees with the under-exploited potential in Kenya

| **Ranking** | ***Scientific name*** | **Common name** | **Selection criteria** | **Reference** |
| --- | --- | --- | --- | --- |
| 1 | *Tamarindus indica L.* | Tamarind | Farmers/consumers’ preferences: Food value, market potential, shelf life, other uses, level of occurrence, germplasm availability | Chikamai et al., 2004 |
| 2 | *Adansonia digitata L.* | Baobab |  |  |
| 3 | *Sclerocarya birrea (A.Rich.) Hochst* | Marula |  |  |
| 4 | *Ziziphus mauritiana Lam.* | Ber |  |  |
| 5 | *Balanites aegyptiaca (L.) Delile* | Desert date |  |  |
|  |  |  |  |  |
| 1 | *Tamarindus indica L.* | Tamarind | Participatory species priority setting | Teklehaimanot 2005, Wanjira 2017 |
| 2 | *Adansonia digitata L.* | Baobab |  |  |
| 3 | *Sclerocarya birrea (A. Rich.) Hochst.* | Marula |  |  |
| 4 | *Ziziphus mauritiana Lam.* | Ber |  |  |
| 5 | *Balanites aegyptiaca (L.) Delile* | Desert date |  |  |
|  |  |  |  |  |
| 1 | *Tamarindus indica L.* | Tamarind | Field appraisal -> Institutional research interest criteria: Research ability, expected adoption, non-financial factors | Tahir and Bashir 2006 |
| 2 | *Adansonia digitata L.* | Baobab |  |  |
| 3 | *Balanites aegyptiaca (L.) Delile* | Desert date |  |  |
| 4 | *Ziziphus mauritiana Lam.* | Ber |  |  |
| 5 | *Vitex doniana Sweet* |  |  |  |
| 6 | *Berchemia discolor (Klotzsch) Hemsl.* | Bird plum |  |  |
|  |  |  |  |  |
| 1 | *Tamarindus indica L.* | Tamarind | General use, food value, economic value | Muok 2019 |
| 2 | *Adansonia digitata L.* | Baobab |  |  |
| 3 | *Ximenia americana L.* |  |  |  |
| 4 | *Carissa spinarum L.* |  |  |  |
| 5 | *Ancylobothrys tayloris (Stapf) Pichon* |  |  |  |
| 6 | *Ziziphus mauritiana Lam.* | Ber |  |  |
| 7 | *Dialium orientale Baker.f.* |  |  |  |
|  |  |  |  |  |
| 1 | *Tamarindus indica L.* | Tamarind | Potential for production, value addition, marketing and economic contribution | Muga et al., 2016 |
| 2 | *Adansonia digitata L.* | Baobab |  |  |
| 3 | *Ximenia americana L.* |  |  |  |
| 4 | *Carissa spinarum L.* | Bush plum |  |  |
| 5 | *Ancylobothrys tayloris (Stapf) Pichon* |  |  |  |
| 6 | *Ziziphus mauritiana Lam.* | Ber |  |  |
| 7 | *Dialium orientale Baker.f.* |  |  |  |
|  |  |  |  |  |
| 1 | *Tamarindus indica L.* | Tamarind | Community participatory setting: preliminary selection | Teklehaymanot 2010 |
| 2 | *Adansonia digitata L.* | Baobab |  |  |
| 3 | *Sclerocarya birrea (A. Rich.) Hochst.* | Marula |  |  |
| 4 | *Ziziphus mauritiana Lam.* | Ber |  |  |
| 5 | *Balanites aegyptiaca (L.) Delile* | Desert date |  |  |
| 6 | *Vitex payos (Lour.) Merr.* | Chocolate Berry |  |  |
| 7 | *Berchemia discolor (Klotzsch) Hemsl.* | Bird plum |  |  |
| 8 | *Carissa spinarum L.* | Bush plum |  |  |
|  |  |  |  |  |
| 1 | *Tamarindus indica L.* | Tamarind | Farmers/consumers’ preferences: Food value, market potential, shelf life, other uses, level of occurrence, germplasm availability | Tahir and Bashir 2006 |
| 2 | *Adansonia digitata L.* | Baobab |  |  |
| 3 | *Balanites aegyptiaca (L.) Delile* | Desert date |  |  |
| 4 | *Berchemia discolor (Klotzsch) Hemsl.* | Bird plum |  |  |
| 5 | *Ziziphus mauritiana Lam.* | Ber |  |  |
| 6 | *Vitex doniana Sweet* |  |  |  |
| 7 | *Saba comorensis (Bojer ex A.DC.) Pichon* |  |  |  |
| 8 | *Lannea alata (Engl.) Engl.* |  |  |  |
| 9 | *Sclerocarya birrea (A.Rich.) Hochst* | Marula |  |  |
| 10 | *Carissa spinarum L.* | Bush plum |  |  |

**Appendix 2**: Priority ranking of traditional fruit trees with their under-exploited potential in Ethiopia

| **Ranking** | ***Scientific name*** | **Common name** | **Selection criteria** | **Reference** |
| --- | --- | --- | --- | --- |
| 1 | *Ziziphus spina-christi (L.) Desf.* | Jujube | Occurrence in quantity, availability during famine, palatability, medicinal, market values | Pauline and Linus 2004, Bhag 2007, Weldekidan et al., 2017 |
| 2 | *Cordia africana Lam.* |  |  |  |
| 3 | *Balanites aegyptiaca (L.) Delile* | Desert date |  |  |
|  |  |  |  |  |
| 1 | *Balanites aegyptiaca (L.) Delile* | Desert date | Food/nutritional value, socio-economic importance, market potential, value addition potential, other uses | Chikamai et al., 2004, Tahir and Bashir 2006 |
| 2 | *Cordeauxia edulis Hemsl.* | Yeheb |  |  |
| 3 | *Mimusops kummel A. DC.* |  |  |  |
| 4 | *Sclerocarya birrea (A. Rich.) Hochst.* | Marula |  |  |
| 5 | *Vitellaria paradoxa Gaertn.* | Shea |  |  |
|  |  |  |  |  |

**Appendix 3**: Big 5 priority traditional fruit trees with their under-exploited potential regions in Africa

| **Region** | **Ranking** | ***Scientific name*** | **Common name** | **Selection criteria** | **Reference** |
| --- | --- | --- | --- | --- | --- |
| East Africa (Ethiopia, Kenya, Sudan, Uganda, Tanzania) | 1 | *Adansonia digitata L.* | Baobab | Priority setting exercises: Field  surveys based on farmers’ preferences and market orientation | Chikamai et al., 2005, Jama et al., 2007, Teklehaimanot 2007, Akinnifesi et al., 2008 |
|  | 2 | *Tamarindus indica L.* | Tamarind |  |  |
|  | 3 | *Ziziphus mauritiana Lam.* | Ber |  |  |
|  | 4 | *Sclerocarya birrea (A. Rich.) Hochst.* | Marula |  |  |
|  | 5 | *Balanites aegyptiaca (L.) Delile* | Desert date |  |  |
|  |  |  |  |  |  |
| Sahelian zone (Seneghal, Mali, Niger, Burkina Faso) | 1 | *Adansonia digitata L.* | Baobab | Priority setting exercises: Field surveys based on quantitative descriptors of variation in indigenous fruit and nut traits - economic importance, visual, organoleptic, and nutritional traits | Franzel et al., 2007, Bounkoungou et al., 1998 |
|  | 2 | *Tamarindus indica L.* | Tamarind |  |  |
|  | 3 | *Vitellaria paradoxa C.F.Gaertn.* | Shea |  |  |
|  | 4 | *Ziziphus spina-christi (L.) Desf.* | Jujube |  |  |
|  | 5 | *Parkia biglobosa (Jacq.) G.Don* | African locust bean |  |  |
|  |  |  |  |  |  |
| West Africa (Ghana, Nigeria, Cameroon) | 1 | *Irvingia gabonensis (Aubry-Lecomte ex O'Rorke) Baill.* | Wild mango | Priority setting exercises: Field surveys based on quantitative descriptors of variation in indigenous fruit and nut traits - economic importance, visual, organoleptic, and nutritional traits | Franzel et al., 2007, |
|  | 2 | *Dacryodes edulis (G.Don) H.J.Lam* | African plum |  |  |
|  | 3 | *Chrysophyllum albidum G.Don* | Star apple |  |  |
|  | 4 | *Garcinia kola Heckel* | Bitter cola |  |  |
|  | 5 | *Cola nitida (Vent.) Schott & Endl.* |  |  |  |
|  |  |  |  |  |  |
| Southern Africa (Malawi, Zambia, Zimbabwe, Tanzania, Mozambique) | 1 | *Uapaca kirkiana Müll.Arg.* | Wild loquat | Priority setting exercises: Field surveys; Participatory Rural Appraisal (PRA) selection; based on market-oriented ideotype products | Maghembe et al., 1998, Akinnifesi et al., 2008 |
|  | 2 | *Strychnos cocculoides Baker* | Wild orange |  |  |
|  | 3 | *Parinari curatellifolia Planch. ex Benth.* | Maula |  |  |
|  | 4 | *Ziziphus spina-christi (L.) Desf.* | Jujube |  |  |
|  | 5 | *Adansonia digitata L.* | Baobab |  |  |
|  |  |  |  |  |  |
| Sahel, South, East Africa | Not ranked | *Adansonia digitata L.* | Baobab | Big five species  based on market extent and preference |  |
| East and West Africa |  | *Balanites aegyptiaca (L.) Delile* | Desert date |  |  |
| South and East Africa |  | *Sclerocarya birrea (A. Rich.) Hochst.* | Marula |  |  |
| South and East Africa |  | *Tamarindus indica L.* | Tamarind |  |  |
| Sahel, South, East Africa |  | *Ziziphus spina-christi (L.) Desf.* | Jujube |  |  |
|  |  |  |  |  |  |
| Africa | Not ranked | *Sclerocarya birrea (A. Rich.) Hochst.* | Marula | Well adapted to arid and semi arid areas | Jama et al., 2007 |
|  |  | *Tamarindus indica L.* | Tamarind |  |  |
|  |  | *Adansonia digitata L.* | Baobab |  |  |
|  |  | *Ziziphus mauritiana Lam.* | Ber |  |  |

**Appendix 4**: Nutrition, income, and ecosystem resilience potential of underutilized and neglected priority fruit trees

| **Fruit tree** | ***Multiple uses** | **Nutrient value** | **Market prospects** | **Ecosystem resilience** | **References** |
| --- | --- | --- | --- | --- | --- |
| *Tamarindus indica L.* (Tamarind) | Food, income, fodder, medicines, food preservatives, wood, fuelwood | proteins, carbohydrates, fibre, vitamin C and B (1, 2, 3), Fe | Fruit pulp is the most valued product | Well adapted to arid and semi-arid areas, drought-tolerant, nitrogen fixing, provides mulch, weed control | Tahir and Bashir 2006, Jama et al., 2007, Van der Stege et al., 2011, Kehlenbeck et al., 2013, Wanjira 2017, Kidaha et al. 2017, Leakey et al., 2022 |
| *Balanites aegyptiaca (L.) Delile* (Desert date) | Food, income, fodder, medicines, insecticide, cosmetics, bio-diesel, wood, fuelwood | protein, fats, vitamin A, minerals | Local market for leaves, fruits, nuts. International market for drugs manufacturing | Found in drylands and very resistant to drought, make very good mulch, nitrogen fixing, highly versatile to Sahelian soil | Tahir and Bashir 2006, Okia 2010, Sagna, et al., 2014, Wanjira 2017, Achaglinkame et al., 2019 |
| *Adansonia digitata L.* (Baobab) | Food, income, fodder, medicines, insecticide, soap making | Vitamin A, C, E, B1, B2, B3, protein, carbohydrate, Fe, Ca, Mg, Zn, P, Kn fiber, ß carotene, amino acids | Every part of the tree is traded and various products approved as ‘novel food’ by European Commission | Drought-tolerant, adaptable to adverse climatic conditions, fertilizer | Jama et al., 2007, Kehlenbeck et al., 2013, Bayala et al. 2014, Boedecker et al. 2014, Chivandi et al. 2015, Wanjira 2017, Vinceti et al. 2018, Akinola et al., 2020, Leakey et al., 2022 |
| *Sclerocarya birrea (A. Rich.) Hochst.* (Marula) | Food, income, fodder, medicines, insecticide, wood, fuelwood | Vitamin A, C, B1, fat, protein, lipids, citric, acid, malic acids, P, Cu, Zn, Ca, Mg, K | Cosmetics industry, biodiesel, food industry | Adapted to arid and semi-arid areas, well adapted to shallow inherently infertile soils | Hall et al., 2002, Tahir and Bashir 2006, Jama et al., 2007, Kehlenbeck et al., 2013, Wanjira 2017, Leakey et al., 2022 |
| *Ziziphus spina-christi (L.) Desf.* (Jujube) | Food, income, fodder, medicines, nematode control, wood, fuelwood | Ca, K, Fe, Cu, Zn | Food industry | Indigenous to dry, low-rainfall and high-temperature areas, drought tolerant and very resistant to heat wave, increase available soil phosphorous | Mokria et al., 2022, Leakey et al., 2022 |

*The multiple uses list is random and not based on ranking of importance nor is it universal across African regions and countries nor within countries.

**Appendix 5**: Nutrition, income, and ecosystem resilience potential of underutilized and neglected leafy vegetables

| **Leafy vegetable** | ***Multiple uses** | **Nutrient value** | **Market prospects** | **Ecosystem resilience** | **References** |
| --- | --- | --- | --- | --- | --- |
| *Amaranthus blitum L.* (Amaranthus) | Food, medicines, beer, fonder, laundry starch, cosmetics, paper coatings, films, dye | Leaves: A, C, B1. Ca, Fe, carotene, folate, vitamin C. Seed: protein (lysine and methionine) fiber, K, Ca, P, vitamin A, C | Seed flour for baking industry - gluten free. Seeds malted for beer | Adapted to different agro-climatic conditions; heat, drought, and pest tolerant and grow in nutrient-poor soil | Calzetta et al., 2000, Park et al., 2020, Ogwu 2020 |
| *Cleome gynandra L.* (Spiderplant) | Food, medicines, insecticidal, fodder, insecticidal and anti-tick properties | Vitamin A, C, E, Ca, Fe, Zn, Mg, β-carotene, protein | Grows very fast; ready rural and urban market, only vegetable available during relish-gap period | Tolerates high and low temperatures, drought; pest resistance; requires fertile soils | Chweya and Nameus, 1997, Van den Heever and Venter, 2007, Onyango et al., 2013, Chataika et al., 2022 |
| *Corchorus olitorius L./tricularis L.*  (Jute mallow) | Food, medicines, cosmetic, packaging fibre, soap, waxes, paper making | Vitamin A, C, E, K, Ca, Mg, Fe, β-carotene, protein | High market value, consumers’ preference, and nutritional value | Available when no other foliage crops can grow; resistant to diseases and pests, adapted to various environments | Choudhary et al., 2013, Mukul 2022 |
| *Crotalaria ochroleuca G.Don/brevidens Benth.* (Slenderleaf) | Food, fodder, medicines, green manure, soil fertility, fibre, cover crop, striga and nematode control, insect repellent, ornamental plant | Vitamin C, β-carotene, B1, B2, B3, protein, Fe, Ca | Demand increase in local or regional markets | Tolerate rather dry conditions; planted during short rainy season; nitrogen-fixing abilities; nematode control | Kullaya et al., 1998, Sikuku et al., 2013, Okelo et al., 2021, Muli et al., 2021 |
| *Launaea cornuta (Hochst. ex Oliv. & Hiern) C.Jeffrey.*  (Bitter Lettuce) | Food, medicines, fodder, insecticidal | Protein, crude fibre, vitamin C, Na, K, Ca, Fe, P | Ready urban market; local availability, growing naturally, low input | Prefers sandy soils in relatively dry localities; threatened by genetic erosion | Ndossi and Sreeramulu 1991, Ambajo and Matheka 2016, Onyancha 2015, Fashir 2015, Akimat et al., 2021 |

*The multiple uses list is random and not based on ranking of importance nor is it universal across African regions and countries nor within countries.

**Appendix 6**: Nutrition, income, and ecosystem resilience potential of underutilized and neglected long-life cycle pulses

| **Leafy vegetable** | ***Multiple uses** | **Nutrient value** | **Market prospects** | **Ecosystem resilience** | **References** |
| --- | --- | --- | --- | --- | --- |
| *Amaranthus blitum L.* (Amaranthus) | Food, medicines, beer, fonder, laundry starch, cosmetics, paper coatings, films, dye | Leaves: A, C, B1. Ca, Fe, carotene, folate, vitamin C. Seed: protein (lysine and methionine) fiber, K, Ca, P, vitamin A, C | Seed flour for baking industry - gluten free. Seeds malted for beer | Adapted to different agro-climatic conditions; heat, drought, and pest tolerant and grow in nutrient-poor soil | Calzetta et al., 2000, Park et al., 2020, Ogwu 2020 |
| *Cleome gynandra L.* (Spiderplant) | Food, medicines, insecticidal, fodder, insecticidal and anti-tick properties | Vitamin A, C, E, Ca, Fe, Zn, Mg, β-carotene, protein | Grows very fast; ready rural and urban market, only vegetable available during relish-gap period | Tolerates high and low temperatures, drought; pest resistance; requires fertile soils | Chweya and Nameus, 1997, Van den Heever and Venter, 2007, Onyango et al., 2013, Chataika et al., 2022 |
| *Corchorus olitorius L./tricularis L.*  (Jute mallow) | Food, medicines, cosmetic, packaging fibre, soap, waxes, paper making | Vitamin A, C, E, K, Ca, Mg, Fe, β-carotene, protein | High market value, consumers’ preference, and nutritional value | Available when no other foliage crops can grow; resistant to diseases and pests, adapted to various environments | Choudhary et al., 2013, Mukul 2022 |
| *Crotalaria ochroleuca G.Don/brevidens Benth.* (Slenderleaf) | Food, fodder, medicines, green manure, soil fertility, fibre, cover crop, striga and nematode control, insect repellent, ornamental plant | Vitamin C, β-carotene, B1, B2, B3, protein, Fe, Ca | Demand increase in local or regional markets | Tolerate rather dry conditions; planted during short rainy season; nitrogen-fixing abilities; nematode control | Kullaya et al., 1998, Sikuku et al., 2013, Okelo et al., 2021, Muli et al., 2021 |
| *Launaea cornuta (Hochst. ex Oliv. & Hiern) C.Jeffrey.*  (Bitter Lettuce) | Food, medicines, fodder, insecticidal | Protein, crude fibre, vitamin C, Na, K, Ca, Fe, P | Ready urban market; local availability, growing naturally, low input | Prefers sandy soils in relatively dry localities; threatened by genetic erosion | Ndossi and Sreeramulu 1991, Ambajo and Matheka 2016, Onyancha 2015, Fashir 2015, Akimat et al., 2021 |

*The multiple uses list is random and not based on ranking of importance nor is it universal across African regions and countries nor within countries.

**References:**

Achaglinkame, M.A., Aderibigbe, R.O., Hensel, O., Sturm, B. and Korese, J.K. (2019). Nutritional Characteristics of Four Underutilized Edible Wild Fruits of Dietary Interest in Ghana. Foods 8(3): 104

Akimat, E., Omwenga, G., Moriasi, G., Ngugi, M. (2021). Antioxidant, Anti-Inflammatory, Acute Oral Toxicity, and Qualitative Phytochemistry of The Aqueous Root Extract of Launaea cornuta (Hochst. Ex Oliv. & Hiern.). Journal of Evidence-Based Integrative Medicine. 26. 1-12. 10.1177/2515690X211064585

Akinnifesi, A.O.C., Sileshi, G.W., Kadzere, I., Akinnifesi, A.I. (2008). Domesticating and Commercializing Indigenous Fruit and Nut Tree Crops for Food Security and Income Generation in Sub-Saharan Africa. Accessed on 25 May 2022 in http://apps.worldagroforestry.org/downloads/Publications/PDFS/pp15397.pdf

Akinola, R., Pereira, L., Mabhaudhi, T., Bruin, F.M., Rusch, L. (2020). A Review of Indigenous Food Crops in Africa and the Implications for more Sustainable and Healthy Food Systems. Sustainability. 12. 3493. 10.3390/su12083493

Ambajo, F., Matheka, J. (2016). Micropropagation of Launaea cornuta - an important indigenous vegetable and medicinal plant. African Journal of Biotechnology. 15. 1726-1730. 10.5897/AJB2016.15260

Bayala, J., Sanou, J., Teklehaimanot, Z., Kalinganire, A., Ouédraogo, S. (2014). Parklands for buffering climate risk and sustaining agricultural production in the Sahel of West Africa. Current Opinion in Environmental Sustainability 6: 28–34

Bhag, M. (2007). Neglected and Underutilized crop genetic resources for sustainable agriculture. Indian Journal Plant Genetic Resource 22:1-16

Boedecker, J., Termote, C., Assogbadjo, A.E., Van Damme, P. and Lachat, C. (2014). Dietary contribution of Wild Edible Plants to women's diets in the buffer zone around the Lama forest, Benin–an underutilized potential. Food Security 6(6): 833–849

Bounkoungou, E.G., Djimde, M., Ayuk, E.T., Zoungrana, I, Tchoundjeu, Z. (1998). Taking Stock of Agroforestry in the Sahel: Harvesting Results for the Future, End of Phase Report: 1989-96, ICRAF, PO Box 30677, Nairobi, Kenya

Calzetta, R.A., Tolaba, M., Suarez, C. (2000). Some physical and thermal characteristics of amaranth starch Algunas propiedades físicas y térmicas del almidón de amaranto. Food Sci. Technol. Int., 6:371–378. doi: 10.1177/108201320000600503

Chataika, B., Akundabweni, L., Sibiya, J., Achigan-Dako, E., Sogbohossou, D., Kwapata, K., Awala, S. (2022). Major Production Constraints and Spider Plant [Gynandropsis gynandra (L.) Briq.] Traits Preferences Amongst Smallholder Farmers of Northern Namibia and Central Malawi. Frontiers in Sustainable Food Systems. 6. 10.3389/fsufs.2022.831821

Chikamai, B., Eyog-Matig, O., Mbogga, M. (2004) Review and appraisal on the status of indigenous fruits in Eastern Africa, A report prepared for IPGRI-SAFORGEN in the framework of AFREA/FORNESSA, IPGRI (International Plant Genetic Resources Institute) SSA, Nairobi, Kenya

Chikamai, B., Eyog-Matig, O., Kweka D. (2005) Regional Consultation on Indigenous Fruit Trees in Eastern Africa. Kenya Forestry Research Institute, Nairobi

Chivandi, E., Mukonowenzou, N., Nyakudya, T., Erlwanger, K.H. (2015). Potential of indigenous fruit-bearing trees to curb malnutrition, improve household food security, income and community health in Sub-Saharan Africa: A review. Food Research International 76: 980–985

Choudhary, S., Sharma, H., Karmakar, P., Arroju, A., Saha, A., Hazra, P., Mahapatra, B. (2013). Nutritional profile of cultivated and wild jute (Corchorus) species. Australian Journal of Crop Science. 7. 1973-1982

Chweya, J., A. Nameus A.M. (1997). Cat’s whiskers. Cleome gynandra L. Promoting the conservation and use of underutilized and neglected crops. 11. Institute of Plant Genetics and Crop Plant Research, Gatersleben/International Plant Genetic Resources Institute, Rome, Italy

Fashir, G. (2015). Assessment the Consumption of Sonchus cornutus (Hochst) in Khartoum State, Sudan. Accessed on 23 September 2022 at https://www.ijcmas.com/vol-4-6/Galal%20Abas%20Fashir,%20et%20al.pdf

Franzel, S., Akinnifesi, F.K., Ham, C. (2007). Setting priorities among indigenous fruit species: Examples from three regions in Africa. In: Indigenous Fruit Trees in the Tropics: Domestication, Utilization and Commercialization. Akinnifesi, F.K., R.R.B. Leakey, O.C. Ajayi, G. Sileshi, Z. Tchoundjeu, P. Matakala and F.R. Kwesiga (eds.). World Agroforestry Centre: Nairobi. CAB International Publishing, Wallingford, UK

Hall, J.B., O’Brien, E.M., Sinclair, F. (2002). Sclerocarya birrea: a monograph. School of Agricultural and Forest Sciences Publication no. 19, University of Wales, Bangor. UK

Jama, B.A., Mohammed, A.M., Mulaya, J., Njui, A.N. (2007). Comparing the ‘Big Five’: A framework for the sustainable management of indigenous fruit trees in the drylands of East and Central Africa. Ecological Indicators doi:10.1016/j.ecolind.2006.11.009

Muga, M., Chikamai, B., Mayunzu, O., Chiteva, R. (2016). Production, Value Addition, Marketing and Economic Contribution of Non Wood Forest Products from Arid and Semi Arid Lands in Kenya. Kenya Forestry Research Institute (KEFRI). Accessed on 5 September 2022 in https://www.fao.org/3/be720e/be720e.pdf

Mukul, M.M. (2022). Perspective Chapter: Nutraceutical Diversity of Eco-Friendly Jute and Allied Fibre (JAF) Crops in Bangladesh. In R. T. Maia, & M. a. de Araújo (Eds.), Population Genetics. IntechOpen. https://doi.org/10.5772/intechopen.102664

Kehlenbeck, K., Asaa,h E., Jamnadass, R. (2013). Diversity of indigenous fruit trees and their contribution to nutrition and livelihoods in sub-Saharan Africa: Examples from Kenya and Cameroon. ISBN: 978-0-203-12726-1 (ebk)

Kidaha, M., Rimberia, F., Kasili, R., Kariuki, W. (2017). Evaluation of Tamarind (Tamarindus indica) Utilization and Production in Eastern Parts of Kenya. Asian Research Journal of Agriculture. 6. 1-7. 10.9734/ARJA/2017/34705

Kullaya, I.K., Kilasara, M., AuneJ, B. (1998). The potential of marejea (Crotalaria ochroleuca) as green manure in maize production in the Kilimanjaro region of Tanzania. DOI: 10.1111/j.1475-2743.1998.tb00627.x

Leakey, R.R.B., Tientcheu A.M.L., Awazi, N.P., Assogbadjo, A.E., Mabhaudhi, T., Hendre, P.S., Degrande, A., Hlahla, S., Manda, L. (2022). The Future of Food: Domestication and Commercialization of Indigenous Food Crops in Africa over the Third Decade (2012–2021). Sustainability, 14, 2355. https://doi.org/10.3390/su14042355

Maghembe, J.A., Simons, A.J., Kwesiga, F., Rarieya, M. (1998). Selecting Indigenous Trees for Domestication in Southern Africa: Priority Setting with Farmers in Malawi, Tanzania, Zambia and Zimbabwe. ICRAF, Nairobi, Kenya. 94pp

Mokria, M., Gebretsadik, Y., Birhane, E., McMullin, S., Ngethe, E., Hadgu, K.M., Hagazi, N., Tewolde-Berhan, S. (2022). Nutritional and ecoclimatic importance of indigenous and naturalized wild edible plant species in Ethiopia. Food Chemistry: Molecular Sciences 4(30 July):100084

Muli, J., Neondo, J., Kamau, P., Odari, E., Budambula, N. (2021). Phenomic characterization of Crotalaria germplasm for crop improvement. 2. 10.1186/s43170-021-00031-0

Muok, B.O. (2019). Potentials and Utilization of Indigenous Fruit Trees for Food and Nutrition Security in East Africa. DOI: 10.5281/zenodo.2583891

Ndossi, G.D., Sreeramulu, N. (1991). Chemical studies on the nutritional value of launaea cornuta a wild leafy vegetable. Journal of Food Science and Technology (Mysore) 28(3): 183-184

Ogwu, M. (2020). Value of Amaranthus [L.] Species in Nigeria. 10.5772/intechopen.86990

Okelo, B., Omami, E., Ngode, L. (2021). Determination of the Effect of Harvesting Interval on the Growth and Leaf Yield of Rattlebox (Crotalaria Ochroleuca). African Journal of Education,Science and Technology, 6(3), Pg 45-63. Retrieved from https://ajest.info/index.php/ajest/article/view/566

Okia, C.A. (2010). Balanites aegyptiaca: A resource for Improving Nutrition and Income of Dryland Communities in Uganda. Accessed on 25 May 2022 in https://core.ac.uk/download/pdf/228909731.pdf

Onyancha, J. (2015). Phytochemical screening and cytotoxicity evaluation of Launaea Cornuta H. (Asteraceae) using brine shrimp. Merit Research Journal of Medicine and Medical Sciences. 4. 116-120

Onyango, C., Kunyanga, C., Ontita, E., Narla, R., Kimenju, J. (2013). Current status on production and utilization of spider plant (Cleome gynandra L.) an underutilized leafy vegetable in Kenya. Genetic Resources and Crop Evolution. 60. 2183-2189. 10.1007/s10722-013-0036-7

Park, S.J., Sharma, A., Lee, H.J. (2020). A Review of Recent Studies on the Antioxidant Activities of a Third-Millennium Food: Amaranthus spp. Antioxidants. 9. 1236. 10.3390/antiox9121236

Pauline, M., Linus, W. (2004): Status of indigenous fruits in Kenya. In: Chikamai, B.et al (eds.) Review and appraisal on the Status of Indigenous Fruits in Eastern Africa. A synthesis report for IPGRI-SAFOREGEN

Sagna, M., Niang, K., Guisse, A., Goffner, D. (2014). Balanites aegyptiaca (L.) Delile: Geographical distribution and ethnobotanical knowledge by local populations in the ferlo (North Senegal). Biotechnology, Agronomy and Society and Environment. 18. 503-511

Sikuku, P., Musyimi, P. Kariuki, S., Okello, S. (2013). Responses of slenderleaf rattlebox (Crotalaria ochroleuca) to water deficit. Journal of Biodiversity and Environmental Sciences (JBES). 3. 245-252

Tahir, El., Bashir, A. (2006). Review and Appraisal on the Status of Indigenous Fruits in Eastern Africa A report prepared for IPGRI-SAFORGEN in the framework of AFREA/FORNESSA FORNESSA-AFREA Logo

Teklehaimanot, Z. (2005) Indigenous fruit trees of Eastern Africa, The Leverhulme Trust: a Study Abroad Fellowship report, University of Wales, Bangor, Uk

Teklehaimanot, Z. (2007). The role of indigenous fruit trees in sustainable dryland agriculture in Eastern Africa. Indigenous Fruit Trees in the Tropics: Domestication, Utillization and Commercialization. 204-223

Teklehaimanot, T., Giday, M. (2010). Ethnobotanical study of wildedible plants of Kara and kwego semi-pastoralist people in lower Omo River Valley, Debub Omo Zone SNNPR, Ethiopia. Journal of Ethnobilogical and Ethnomedicine, 6:23

Van den Heever, E., Venter, S. (2007). Nutritional and medicinal properties of Cleome gynandra. Acta Horticulturae. 752. 127-130. 10.17660/ActaHortic.2007.752.17

Van der Stege, C., Prehsler, S., Hartl, A., Vogl, C.R. (2011). Tamarind (Tamarindus indica L.) in the traditional West African diet: Not just a famine food. Fruits. 66. 171-185. 10.1051/fruits/2011025

Vinceti, B., Termote, C., Thiombiano, N., Agúndez, D., Lamien, N. (2018). Food tree species consumed during periods of food shortage in Burkina Faso and their threats. Forest Systems 27(2): e006

Wanjira, E. (2017). Wild edible indigenous fruit tree species in East Africa’s drylands: A coping mechanism for vulnerable farmers and pastoralists against drought and hunger. Accessed on 6 September 2022 in https://www.researchgate.net/publication/339686255_The_Contributions_of_Wild_Tree_Resources_to_Food_and_Nutrition_Security_in_Sub-Saharan_African_Drylands_A_Review_of_the_Pathways_and_Beneficiaries

Weldekidan, N., Hruy, G., Gebrelibanos, T. (2017). Potentials and constraints of under-utilized tree fruits and vegetables in Tigray, Northern Ethiopia. 7. 664-674
